# Acupuncture-Induced Pneumothorax Following Thoracic Acupuncture in a Patient With Ehlers-Danlos Syndrome: A Case Report

**DOI:** 10.7759/cureus.115879

**Published:** 2026-09-07

**Authors:** Muhammad Zulqarnain, Amjad Irfan

**Affiliations:** 1 Acute Medicine, Whittington Hospital, London, GBR; 2 Neurology, Letterkenny University Hospital, Letterkenny, IRL

**Keywords:** acupuncture, case report, cluster headache, ehlers-danlos syndrome, pleural disease, pneumothorax

## Abstract

Acupuncture is widely used as an adjunctive therapy for chronic pain and headache disorders and is generally considered a safe procedure when performed by trained practitioners. However, pneumothorax remains one of its most serious, albeit uncommon, complications. Patients with connective tissue disorders, such as Ehlers-Danlos syndrome (EDS), may theoretically be more susceptible to procedure-related complications because of underlying tissue fragility, although reports describing acupuncture-induced pneumothorax in this population are exceptionally rare. We present the case of a young woman with EDS and chronic refractory cluster headaches who developed an iatrogenic left-sided pneumothorax following acupuncture involving the posterior thoracic region. She presented with sudden-onset pleuritic chest pain and progressive dyspnoea approximately three hours after treatment. Chest radiography demonstrated a 2.8-cm left apical pneumothorax. Needle aspiration removed approximately 450 mL of air and resulted in immediate symptomatic improvement, although repeat chest radiography demonstrated minimal reduction in the size of the pneumothorax. Following multidisciplinary discussion, she was managed conservatively with close inpatient observation. Computed tomography excluded underlying bullous lung disease or other structural abnormalities, and serial imaging demonstrated complete spontaneous resolution within 24 hours. This case highlights the importance of considering pneumothorax in patients presenting with respiratory symptoms following thoracic acupuncture and demonstrates that carefully selected, clinically stable patients may be managed conservatively in accordance with contemporary British Thoracic Society guidance.

## Introduction

Acupuncture is one of the most commonly practiced complementary therapies worldwide and is increasingly used as an adjunctive treatment for patients with chronic pain disorders who have persistent symptoms despite conventional medical therapy. It is frequently employed in the management of musculoskeletal pain, migraine, tension-type headache, and refractory cluster headache because of its relatively favourable safety profile and low incidence of serious adverse events. Most complications are minor and self-limiting, including localized pain, bruising, bleeding, and transient dizziness. However, although uncommon, serious complications, such as pneumothorax, cardiac tamponade, infection, and injury to major neurovascular structures, have been reported, particularly when acupuncture is performed over the thoracic or paraspinal regions [1].

Pneumothorax is considered one of the most important potentially life-threatening complications of acupuncture. In a large population-based study from Taiwan involving 5,407,378 acupuncture treatments, the incidence of iatrogenic pneumothorax was 0.87 per 1,000,000 acupuncture treatments overall and 1.75 per 1,000,000 treatments when acupuncture was performed at "at-risk" anatomical sites [2]. Patients usually present within hours of the procedure with pleuritic chest pain, dyspnoea, or chest discomfort, symptoms that may initially be mistaken for expected post-procedural soreness. Consequently, failure to recognize this complication may delay diagnosis and appropriate management. Healthcare professionals should therefore maintain a high index of suspicion and routinely inquire about recent acupuncture in patients presenting with acute pleuritic chest pain or unexplained breathlessness [3].

Ehlers-Danlos syndrome (EDS) comprises a heterogeneous group of inherited connective tissue disorders caused by abnormalities in collagen synthesis and extracellular matrix structure. Depending on the subtype, affected individuals may exhibit generalized joint hypermobility, skin hyperextensibility, poor wound healing, vascular fragility, and increased susceptibility to tissue injury. Pulmonary manifestations are uncommon in most forms of EDS, although spontaneous pneumothorax has been reported, particularly in vascular EDS, where connective tissue fragility predisposes to pleural and pulmonary complications [4]. In the present case, the patient had classical EDS. Whether patients with classical EDS or other non-vascular EDS subtypes have increased susceptibility to procedure-related complications remains uncertain because available evidence is limited.

Despite the widespread use of acupuncture, reports of acupuncture-induced pneumothorax in patients with EDS are exceedingly rare. Furthermore, management of pneumothorax in these patients may be challenging because the potential benefits of invasive pleural procedures must be balanced against concerns regarding tissue fragility and possible procedural complications. We present the case of a young woman with EDS who developed a symptomatic left-sided pneumothorax shortly after acupuncture for chronic refractory cluster headaches. This case highlights the importance of recognizing pneumothorax as a rare but serious complication of thoracic acupuncture, particularly in patients with underlying connective tissue disorders, and emphasizes the role of individualized management and shared decision-making.

## Case presentation

A young woman with a known history of EDS and chronic refractory cluster headaches presented to the emergency department with sudden-onset left-sided pleuritic chest pain and progressively worsening shortness of breath.

She reported experiencing debilitating daily cluster headaches despite treatment with subcutaneous sumatriptan and multiple analgesic medications. Owing to persistent symptoms and inadequate relief with pharmacological therapy, she sought acupuncture treatment involving multiple needle insertions over the posterior upper thorax and paraspinal region. The acupuncture session lasted approximately 30-45 minutes. She described the acupuncture needles as much thinner than an intravenous cannula but considerably longer, and reported a sensation that the needles were being inserted deeply into her back.

Immediately after the acupuncture session, she experienced localized discomfort at the needle insertion sites, which she initially attributed to the procedure. She took paracetamol at home with minimal improvement. Approximately three hours later, she developed sudden-onset left-sided pleuritic chest pain associated with progressively worsening dyspnoea. The pain was markedly aggravated by deep inspiration and became sufficiently severe to prompt presentation to the emergency department.

On arrival, her blood pressure was 110/70 mmHg, with a heart rate of 76 beats/minute, a respiratory rate of 26 breaths/minute, oxygen saturation of 96% on room air, temperature of 36.7°C, and capillary blood glucose of 6.8 mmol/L.

Respiratory examination demonstrated reduced air entry over the left upper lung field with hyperresonance to percussion. There was no tracheal deviation or subcutaneous emphysema. Cardiovascular examination revealed normal heart sounds without murmurs. The remainder of the systemic examination was unremarkable.

Given her diagnosis of EDS, further history explored previous disease-related complications, including spontaneous pneumothoraces, vascular events, recurrent joint dislocations, and previous thoracic surgery. She denied any previous thoracic or vascular complications and had no prior history of pneumothorax.

A 12-lead electrocardiogram demonstrated normal sinus rhythm at 76 beats/minute without acute ischaemic changes (Figure 1).

**Figure 1:**
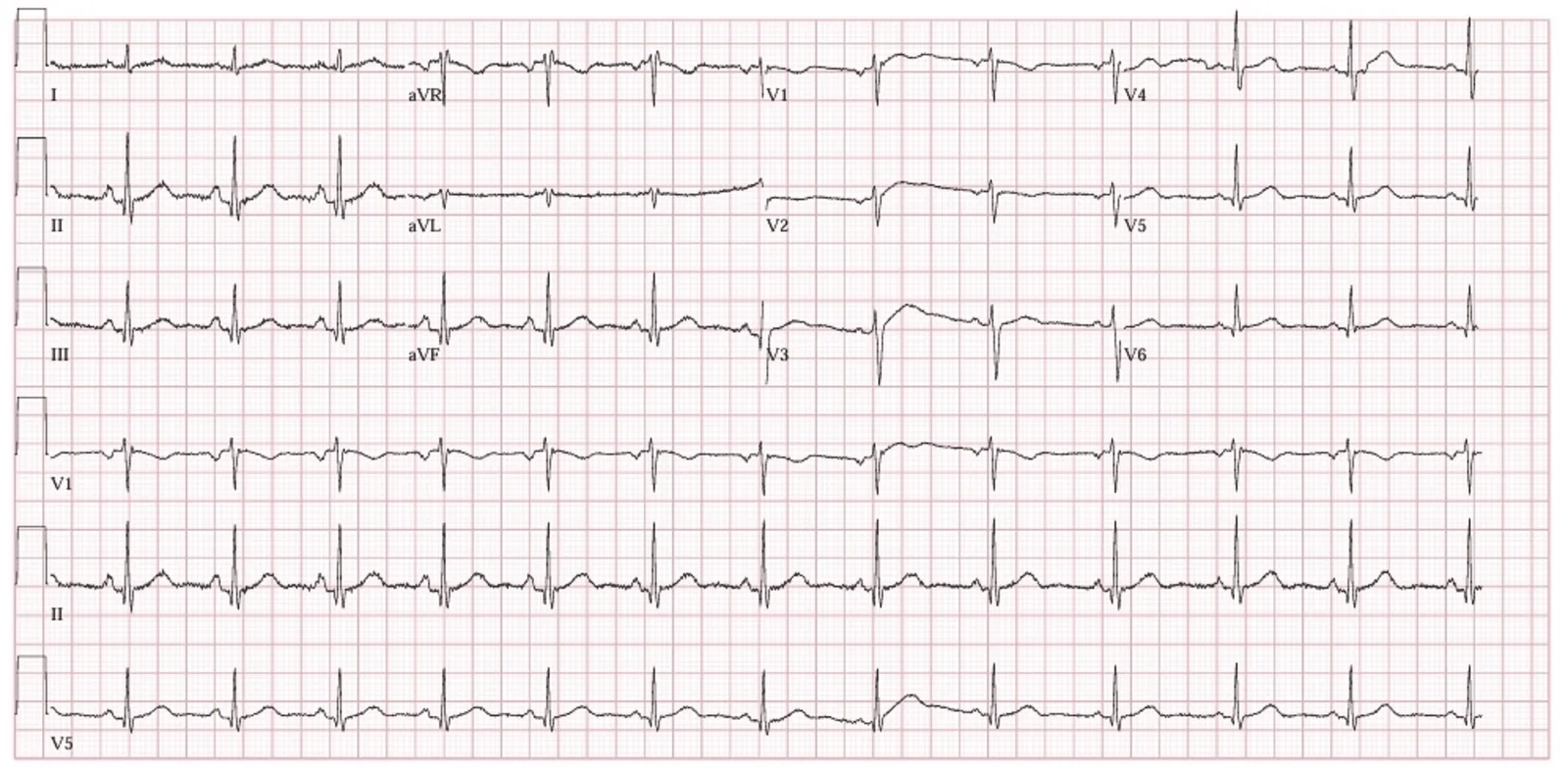
Normal electrocardiogram (ECG) A 12-lead ECG demonstrating a normal sinus rhythm with normal cardiac axis and intervals, without significant ST-segment or T-wave abnormalities.

Laboratory investigations, including complete blood count, renal function, electrolytes, C-reactive protein, and high-sensitivity cardiac troponin T, were all within normal reference ranges (Table 1).

**Table 1:** Initial laboratory investigations eGFR (CKD-EPI): estimated glomerular filtration rate (chronic kidney disease-epidemiology collaboration).

Investigation	Results	Reference range
White blood cell count	5.30 × 10⁹/L	3.0-10.0 × 10⁹/L
Haemoglobin	142 g/L	130-170 g/L
Platelets	234 × 10⁹/L	150-400 × 10⁹/L
Urea	3.4 mmol/L	2.5-7.8 mmol/L
Creatinine	70 µmol/L	59-104 µmol/L
eGFR (CKD-EPI)	>90 mL/min/1.73 m²	-
Sodium	141 mmol/L	133-146 mmol/L
Potassium	4.7 mmol/L	3.5-5.3 mmol/L
Total bilirubin	10 µmol/L	0-20 µmol/L
Alanine aminotransferase	44 U/L	10-50 U/L
Alkaline phosphatase	88 U/L	30-130 U/L
Total protein	48 g/L	60-80 g/L
Albumin	35 g/L	35-50 g/L
Creatine kinase	242 U/L	39-308 U/L
Aspartate aminotransferase	35 U/L	10-50 U/L
Cardiac troponin T	10 ng/L	0-13 ng/L
C-Reactive protein	3 mg/L	< 5 mg/L

Chest radiography demonstrated a left apical pneumothorax measuring approximately 2.8 cm with absent peripheral lung markings (Figure 2).

**Figure 2:**
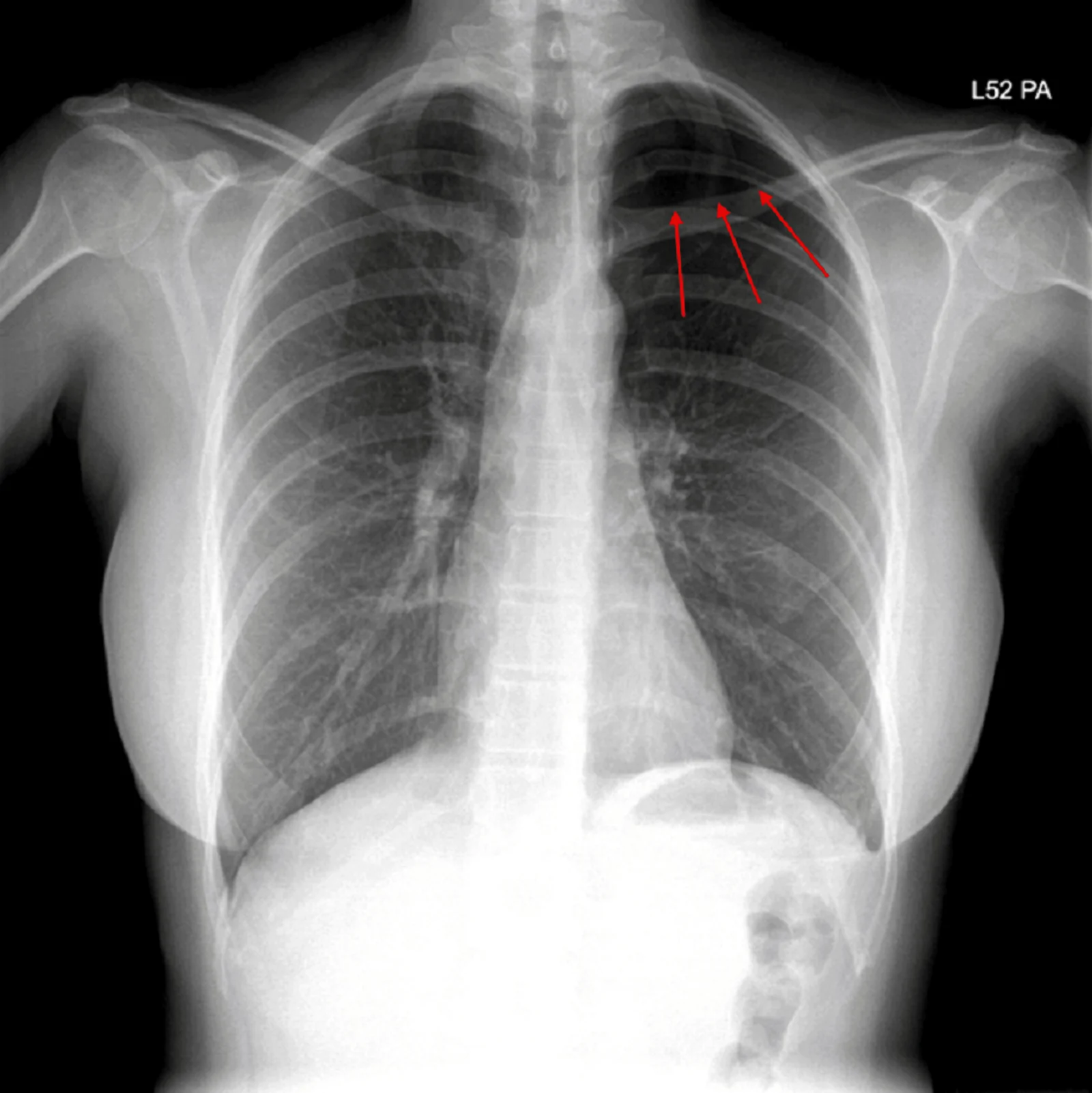
Chest X-ray posteroanterior (PA) view demonstrating a left apical pneumothorax Posteroanterior (PA) chest radiograph demonstrating a left apical pneumothorax with a visible visceral pleural line and the absence of peripheral lung markings, as shown by the red arrows.

As the patient was symptomatic with significant pleuritic chest pain and dyspnoea, immediate needle aspiration was performed using an anterior second intercostal space approach under aseptic conditions. Approximately 450 mL of air was successfully aspirated, resulting in immediate improvement in both breathlessness and pleuritic chest pain. Following aspiration, her observations remained stable, with a respiratory rate of 18 breaths/minute and oxygen saturation of 97% on room air. However, respiratory examination continued to demonstrate reduced air entry over the left apex, and repeat chest radiography demonstrated a persistent left-sided pneumothorax measuring approximately 2.3 cm (Figure 3).

**Figure 3:**
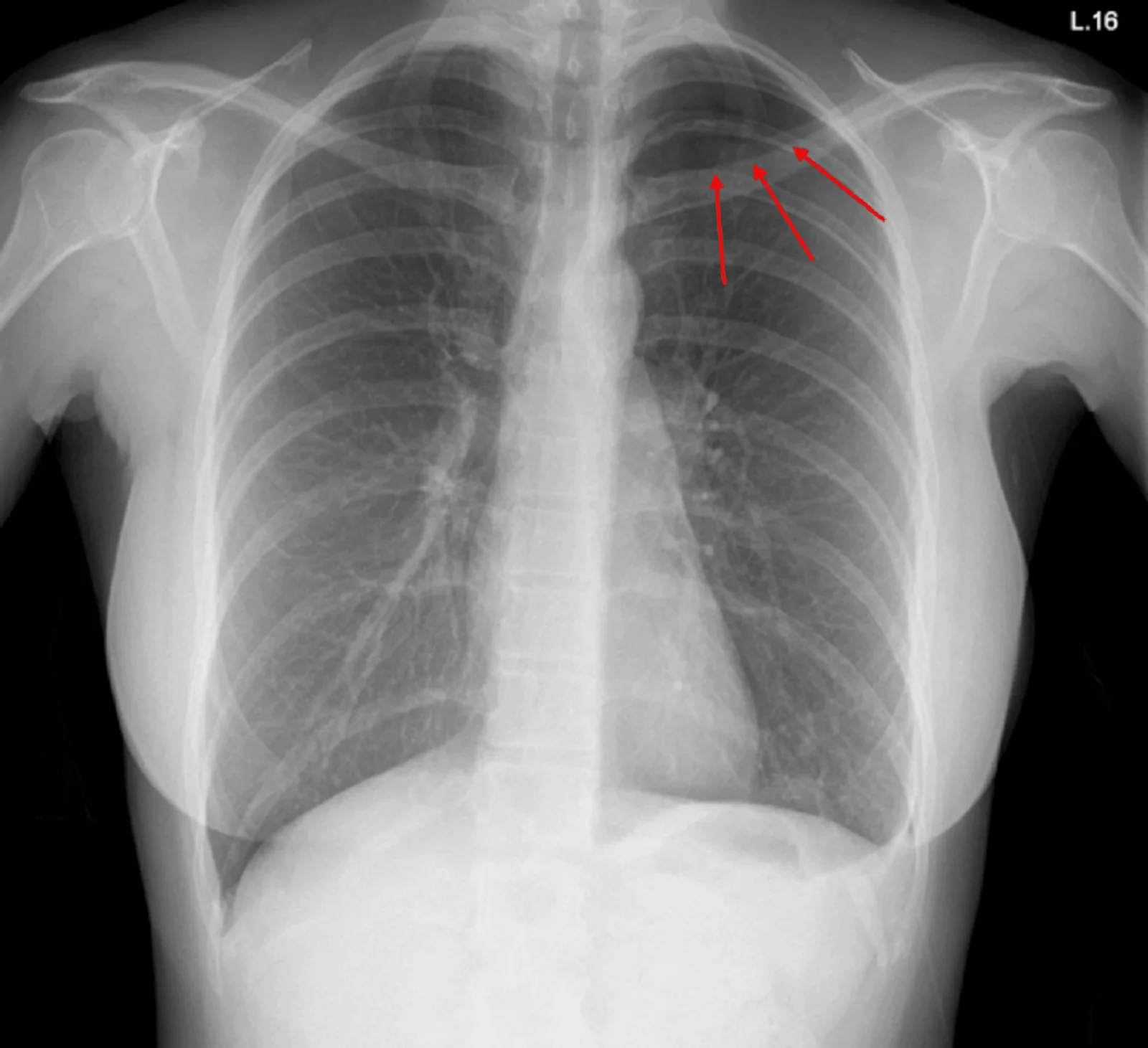
Chest X-ray posteroanterior (PA) view post aspiration Posteroanterior (PA) chest radiograph demonstrating a persistent left apical pneumothorax despite pleural aspiration, as shown by the red arrows.

The respiratory team was consulted for further management. As the patient remained haemodynamically stable and her symptoms had almost completely resolved, both intercostal chest drain insertion and conservative inpatient observation were discussed in detail. Particular consideration was given to her underlying EDS because of the potential procedural risks associated with chest drain insertion. The risks and benefits of both approaches were explained, and following shared decision-making, the patient opted for conservative observation.

Computed tomography of the thorax was subsequently performed to characterise the pneumothorax better and to assess for underlying structural lung abnormalities, including apical blebs, bullae, cystic lung disease, or other pathology that might have predisposed to pneumothorax. The CT scan confirmed the presence of a left-sided pneumothorax but demonstrated no evidence of bullous disease, blebs, emphysema, or other pulmonary abnormalities (Figure 4).

**Figure 4:**
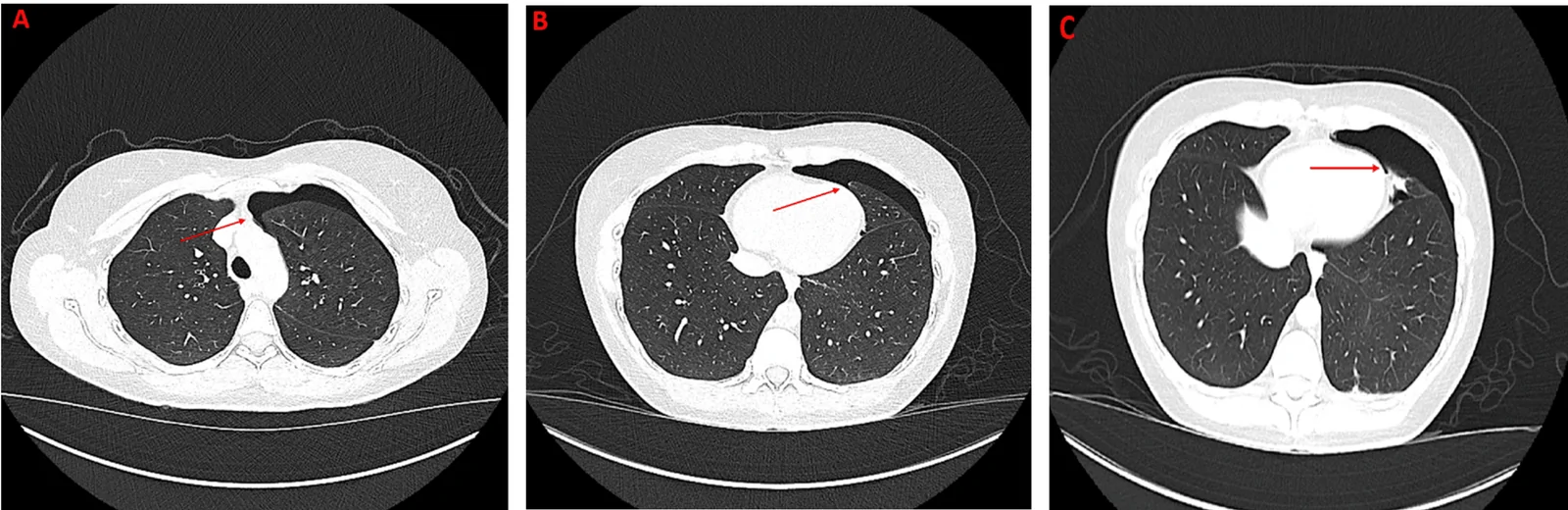
Axial CT scan demonstrating persistent a left apical pneumothorax following aspiration Axial computed tomography (CT) image of the chest demonstrating a persistent left-sided pneumothorax despite pleural aspiration, as shown in images A, B, and C with red arrows.

After 24 hours of observation, the patient was clinically stable, with an oxygen saturation of 97% on room air, a respiratory rate of 18 breaths/min, a blood pressure of 110/78 mmHg, and a heart rate of 79 beats/min. Chest examination revealed equal bilateral air entry throughout both lung fields. Repeat chest radiography demonstrated complete spontaneous resolution of the pneumothorax without any intervention, with no evidence of residual pneumothorax (Figure 5).

**Figure 5:**
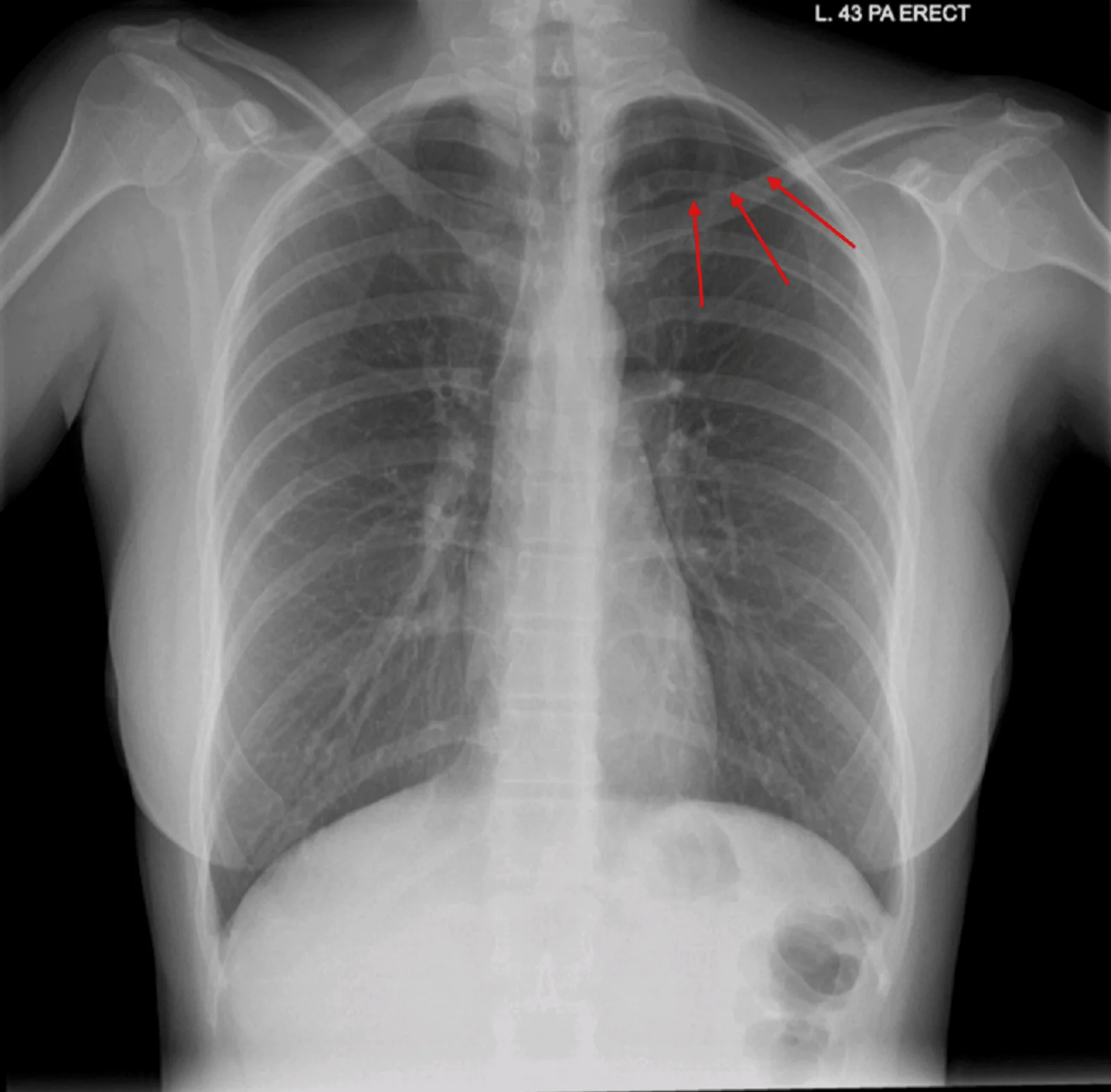
Chest X-ray posteroanterior (PA) view demonstrating complete resolution of the left apical pneumothorax Posteroanterior (PA) chest radiograph demonstrating complete resolution of the left apical pneumothorax with satisfactory re-expansion of the left lung and no residual pleural air, as shown by the red arrows.

She was discharged with advice to avoid future acupuncture involving the thoracic region and was counselled regarding the early recognition of pneumothorax symptoms requiring urgent medical assessment.

## Discussion

Acupuncture is widely regarded as a safe treatment when performed by appropriately trained practitioners, and serious adverse events are uncommon. Large prospective studies have shown that most complications are minor and self-limiting, including local pain, bruising, or transient bleeding. Nevertheless, pneumothorax remains the most frequently reported serious complication associated with acupuncture and has been described in numerous case reports and systematic reviews. Although rare, delayed recognition may result in progressive respiratory compromise or, in severe cases, tension pneumothorax requiring emergency intervention [1,3].

The mechanism of injury involves inadvertent penetration of the parietal pleura during needle insertion, allowing air to enter the pleural cavity. This risk is greatest when acupuncture is performed over the upper posterior thorax, scapular region, or paraspinal muscles because of the close anatomical relationship between the skin surface and pleura. Individual anatomical variation, low body mass index, needle depth, and insertion angle may further increase the likelihood of pleural injury. In the present case, the close temporal relationship between thoracic acupuncture and the onset of pleuritic chest pain strongly supports direct pleural puncture as the underlying mechanism.

Our patient had a known diagnosis of classical EDS but no previous history of spontaneous pneumothorax, thoracic surgery, or underlying pulmonary disease. Computed tomography demonstrated no evidence of apical blebs, bullae, emphysema, cystic lung disease, or other structural abnormalities that could have predisposed to spontaneous pneumothorax. These findings, together with the close temporal relationship between the procedure and symptom onset, support acupuncture as the precipitating event rather than spontaneous rupture of pre-existing pulmonary pathology. Although spontaneous pneumothorax has been reported particularly in vascular EDS, there is very limited literature describing acupuncture-related pneumothorax in patients with EDS. In the present case of classical EDS, connective-tissue fragility could theoretically increase susceptibility to tissue injury during invasive procedures; however, this remains a proposed mechanism rather than an established causal association, and there is currently insufficient evidence to determine whether classical EDS independently increases the risk of pleural injury from acupuncture [4].

Management following unsuccessful needle aspiration required careful consideration. Although repeat chest radiography demonstrated only minimal reduction in pneumothorax size, the patient experienced almost complete resolution of breathlessness and pleuritic chest pain and remained haemodynamically stable. Current British Thoracic Society guidance recommends that management should be individualized according to the patient's clinical condition, symptom burden, physiological stability, and patient preference rather than radiographic appearance alone [5]. Consequently, both chest drain insertion and conservative inpatient observation represented reasonable management options.

The presence of EDS was an important factor during shared decision-making. Although evidence regarding pleural interventions in patients with EDS remains limited, tissue fragility and impaired wound healing may theoretically increase procedural risks. After discussion with the respiratory team, conservative observation was selected because the patient was clinically stable and had experienced significant symptomatic improvement following aspiration. Repeat chest radiography after 24 hours demonstrated complete resolution of the pneumothorax without further intervention. This outcome is consistent with growing evidence supporting conservative management in carefully selected patients with stable pneumothorax [5,6].

This case also highlights the importance of obtaining a thorough procedural history when assessing patients presenting with acute pleuritic chest pain or dyspnoea. Symptoms developing shortly after acupuncture should not be attributed solely to expected post-procedural discomfort, particularly when thoracic needling has been performed. Early chest radiography remains essential for prompt diagnosis and appropriate management.

Acupuncture continues to have an important role as an adjunctive treatment for chronic headache disorders when conventional therapies provide inadequate symptom control [7]. However, clinicians and acupuncture practitioners should remain aware of the rare risk of pneumothorax, particularly in patients with underlying connective tissue disorders. Careful patient selection, appropriate technique, and prompt recognition of post-procedural respiratory symptoms are essential to minimize morbidity. To our knowledge, this represents one of the very few reported cases of acupuncture-induced pneumothorax occurring in a patient with EDS.

## Conclusions

Pneumothorax should be considered in any patient presenting with pleuritic chest pain or dyspnoea following acupuncture involving the thoracic region. Although acupuncture-related pneumothorax is uncommon, prompt recognition and appropriate imaging are essential to avoid delayed diagnosis and potentially life-threatening complications.

This case demonstrates that symptom-guided conservative management with close observation may be a safe alternative to chest drain insertion in carefully selected clinically stable patients, even when repeat imaging demonstrates persistent pneumothorax following aspiration, in accordance with current British Thoracic Society recommendations.
